# Development of novel EST-SSR markers in the macaúba palm (*Acrocomia aculeata*) using transcriptome sequencing and cross-species transferability in Arecaceae species

**DOI:** 10.1186/s12870-018-1509-9

**Published:** 2018-11-12

**Authors:** Bárbara Regina Bazzo, Lucas Miguel de Carvalho, Marcelo Falsarella Carazzolle, Gonçalo Amarante Guimarães Pereira, Carlos Augusto Colombo

**Affiliations:** 1 Institute of Biology, Laboratory of Genomic and Expression, State University of Campinas, Campinas, Brazil; 2 Agronomic Institute of São Paulo (IAC), Genetic Resources Center, Campinas, SP Brazil

**Keywords:** RNA-Seq, Genic molecular markers, Transcript sequences, Genetic diversity, Polymorphism, Macaúba palm

## Abstract

**Background:**

The macaúba palm is a novel feedstock for oil production suitable for multiple uses, including as biodiesel and in the food and cosmetic industries. As an efficient alternative, the macaúba palm has limited genomic resources, particularly expressed sequence tag (EST) markers. We report a comprehensive set of validated EST-simple sequence repeat (SSR) markers by using transcriptome sequencing, its application in genetic diversity analysis and cross transferability in other palm trees with environmental and economic importance.

**Results:**

In this study, a total of 418 EST-SSRs were identified to be unique for one transcript and region; 232 EST-SSRs were selected, with trinucleotide repeats being the most frequent motif, representing 380 (90.9%), followed by composited (4.5%), di- (3.6%), and hexanucleotides (3.6%). A total of 145 EST-SSRs (62.5%) were validated for consistent amplification in seventeen macaúba palm samples, and 100 were determined to be polymorphic with PIC values ranging from 0.25 to 0.77. Genetic diversity analysis was performed with the 20 most informative EST-SSR markers showing a distinct separation of the different groups of macaúba palm. Additionally, these 145 markers were transferred in six other palm species resulting in transferability rates of 99% (144) in *Acrocomia intumescens*, 98% (143) in *Acrocomia totai*, 80.7% (117 EST-EST) in African oil palm (*Elaeis guineensis*) and peach palm (*Bactris gasipaes*) samples, 70% (102) in the juçara palm (*Euterpe edulis*) and 71.7% (104) in the hat palm (*Sabal causiarum*). Analysis of genetic distance showed a high separation in accordance with geographic location, establishing distinct groups by genera.

**Conclusions:**

The EST markers identified in our study are a valuable resource and provide a genomic tool for genetic mapping and further genetic studies, as well as evaluation of co-location between QTLs and functionally associated markers**.**

**Electronic supplementary material:**

The online version of this article (10.1186/s12870-018-1509-9) contains supplementary material, which is available to authorized users.

## Background

The macaúba palm (*Acrocomia aculeata* (Jacq.) Lodd. Ex Mart., 2n = 30) is a native palm tree from America, belonging to the Arecaceae family. It is an arboreous oleiferous species, perennial, heliophilous, monoecious, with a single stem between 4 and 15 m tall and 20–30 cm in diameter [1]. The species is monoecious, and its genome size is 5.81 pg, distributed in 15 pairs of chromosomes (2n = 30) with a base composition of AT = 58.3% [2]. *Acrocomia aculeata* has been documented to inhabit areas from north Florida, Mexico and West Indies to south Paraguay and north Argentina; it is considered the most widespread palm in Brazil, and it can be found especially in the Midwest and Southeast region [3]. It grows naturally in large populations, is adapted to different ecosystems and can be used to rehabilitate degraded pastures or in agroforestry systems.

Its fruit, a product of great economic value, contains a large amount of pulp oil with oil content values up to 75% for the pulp and 65% for oil on the dry basis [4]. It is globose, with a fibrous mesocarp, strongly adhered endocarp, and a large endosperm, with up to four seeds per fruit. Mesocarp oil is rich in oleic acid with high oxidative stability and operability at low temperatures [5, 6]. The oil extracted from the endosperm is rich in short-chained saturated fatty acids, primarily lauric acid, constituting a valuable source for pharmaceutical and cosmetic use [7–9].

The macaúba palm has a great production potential for oil, similar to the African oil palm (*Elaeis guineensis*), whose global production reached 69 million of tons in 2017, representing 34% of global oil production [10]. The productivity of selected native plants of macaúba may reach 5000 kg of oil per ha^− 1^ year^− 1^ [11, 12], making it an inexpensive feedstock for oil production. Moreover, the macaúba palm is considered a novel oil-feedstock crop with a potential role as an environmentally and socially co-beneficial feedstock in South America. Its large-scale cultivation should be conducted in permanent protection areas, pastures, disturbed areas, and tilled land.

Despite its incipient domestication and current use based on its extractive character, macaúba has several uses, with multiple products from its exploitation including food, cosmetics, animal feed, and biofuels [12]. Currently, the renewed interest in this novel feedstock increased commercial interest and can lead to the propagation of plants without agronomic quality, which would render their competitiveness unfeasible.

Precaution should be taken in the use of non-domesticated feedstock species because possible biological variations and genetic diversity is observed in the macaúba in different environments of occurrence [13]. In this context, molecular markers are considered an essential tool to identify and to select superior plants for adoption in large-scale commercial crops, establishment of core collections, creation of seed garden, and initiation of breeding studies [14, 15].

Molecular markers are widely used to track loci and genome regions in several crop-breeding programmes. Furthermore, they can accelerate the generation of new varieties and allow for the connection of phenotypic characters with the genomic loci responsible for them [16, 17]. Molecular marker selection (MMS) is considered a simple and rapid technique, thus accelerating breeding time and developing segregating populations for several generations [16].

Microsatellites or single sequence repeats (SSRs), primarily genic SSRs (or EST-SSRs), are widely employed in palm tree studies with commercial interest [14, 18–21]. In addition to being functional, EST-SSR can lead to a gain or loss of gene function via frameshift mutation or other changes in the amino acid sequence [22]; this is useful for marker-assisted selection, especially when the markers reside in the genes responsible for a phenotypic trait. Assessing the performance and genetic diversity of the natural material is important for understanding the genetic structure and consequently for guiding breeding programmes to develop superior genotypes.

Cross-transferability is a dominant feature of EST-SSR markers among distantly related species and can shed light on the evolution of plant genomes, changes in gene location, and genome organization, whereas the genomic SSRs are not suitable for this purpose. EST-SSR transferability provides a cost-effective source of markers for related species, which is important for taxa with low microsatellite frequencies or for those whose microsatellites are difficult to isolate. Among Arecaceae plants, few species have SSR available, and its cross-transferability is an alternative for species with less information [23, 24].

Despite its increasing promotion in Brazil and the availability of molecular study data, only few SSR markers have been determined to be useful and validated for *A. aculeata*. The present study involves the first transcriptome sequencing of 8 *A. aculeata* tissues using the Illumina Hiseq2500 sequencing platform. The goal of this study was to provide a polymorphic set of genic microsatellite markers, which will allow for the improvement of the understanding of the genetic diversity, genotype characterization and genetic structure of *A. aculeata*, Acrocomia genera, and other environmental and commercial important palm species. Additionally, these markers will be useful in modern *A. aculeata* breeding programmes.

## Results

### Sequencing and reference assembly of Illumina paired-end reads

The cDNA libraries were sequenced using Illumina/HISEQ2000 (Illumina Inc. San Diego, CA, USA), producing millions of 100-bp paired-end reads. For each individual, the sequenced reads from all tissues were grouped and submitted to reference-based transcriptome assembly using the African oil palm genome as a reference. We decided to assemble the individuals separately to avoid the chimeric combination of EST-SSRs that may vary in size among the individuals. The average alignment rate was 71%, ranging from 64.6% (root samples from Amparo and Pedreira) to 80.2% (endosperm sample from Ibituruna). The transcriptome assemblies for the individuals i1, i2 and i3 were performed using vegetative tissues (merging leaves, roots, leaf sheaths and bulbs) that generated 60.323, 61.093 and 61.316 transcript sequences, respectively. The transcriptome assemblies of the female and male flowers resulted in 59.141 and 58.452 transcripts for the individuals i4, i5, and i6, respectively. With respect to fruit libraries (mesocarp and endosperm), the assembly produced 26.447, 28.749, 23.861, 29.847, 26.191, and 26.086 for the individuals i7, i8, i9, i10, i11, and i12, respectively. The minimum average size for all transcripts identified was 200 bp.

### Frequency and distribution of EST-SSR

We identified 85.014 redundant EST-SSRs from 455.051 transcript sequences from all individuals. Among them, 7.492 EST-SSRs were chosen based on the criteria of flanking primers that generate PCR product size ranging from 100 to 500 bp and located inside the same exon. Of these, a total of 418 non-redundant EST-SSRs were selected as representative of each locus, of which sixty-three (15%) were exclusive to vegetative tissues, twenty-three (5.5%) to flower tissues, and twenty-eight (6.7%) to fruit. The number of repeats by SSR motif ranged from 5 to 13 repeats, with 5 and 6 being the most frequent (Table 1). Regarding the location in the African oil palm genome, chromosomes 1 and 2 harboured the majority of the markers, with 38 EST-SSRs in each, followed by chromosome 3 (36 EST-SSRs) and chromosome 4 (23).

**Table 1:** Number and frequency of the 418 EST-SSRs identified by the tissue from which they were derived, number of motif repeats and SSR motif in the macaúba palm transcriptome data set

	Number of EST-SSRs	Frequency (%)
Tissue		
Flower (female and male)	23	5.50
Fruit	28	6.70
Vegetative tissue	63	15.07
Vegetative tissue/flower	60	14.35
Vegetative tissue/fruit	21	5.02
Fruit/flower	19	4.55
Vegetative tissue/flower/fruit	204	48.80
Number of repeats		
5	197	47.13
6	109	26.08
7	48	11.48
8	21	5.02
9	12	2.87
10	2	0.48
11	4	0.96
12	5	1.20
13	1	0.24
SSR Motif		
Dinucleotide	15	3.59
Trinucleotide	380	90.91
Hexanucleotide	15	3.59
Compound	19	4.55

The trinucleotide repeats exhibit the highest frequency of occurrence (380–90.91%), followed by composite repeats, dinucleotides, and hexanucleotides (4.55, 3.59 and 3.59%, respectively) (Table 1). Among the trinucleotide motifs, the most frequent motifs are GAG (7.18%, 30), CCT (5.02%, 21), GCC (4.78%, 20), GGA (4.55%, 19), CAG (3.83%, 16), GGC, AAG, AGG, GCC, CGG, GGT and TCC (see additional file 1). The major dinucleotide EST-SSR motif observed in the macaúba palm is CT/AG, comprising 66% of these motifs.

### Development and validation of genic-SSR markers

To prevent amplicon size deviations, only EST-SSRs inside one exon were chosen for validation. A minimum distance of 1 Mpb between each marker was considered for validation to reduce the chance of linkage equilibrium among the markers. A total of 481 EST-SSR primers flanking unique sequences were designed, which were designated Acro01 to Acro418 (Acro = ‘Acrocomia’).

A subset of 232 EST-SSRs was selected according to the trinucleotide motif, amplicon size (from 100 to 500 bp), and melting temperature of both forward and reverse primers. They were selected to monitor polymorphisms in seventeen samples of macaúba palm from different geographic regions. Additionally, the annotation of these 232 EST-SSRs was performed according to the African oil palm genome.

Of the 232 EST-SSRs tested, 145 (62.5%) were successfully amplified in the genomic DNA, producing clear PCR amplicons with the expected sizes. In total, the 145 EST-SSR markers generated 476 markers bands, and all markers were used for further analysis. The mean number of alleles per loci was 3.28, ranging from 1 to 11 alleles, and 87 (60%) markers had three or more alleles. The effective number of alleles per locus (Ne), expected heterozygosity (He), and observed heterozygosity (Ho) ranged from 1 to 5.02 (Acro205), 0 to 0.828 (Acro201), and 0 to 1.0 (Acro16, Acro64, Acro172), respectively. In addition, Shannon’s information index (I) values ranged from 0 to 1.93 (Acro205), probability of identity (PI) ranged from 0.063 to 1.0, and PIC values ranged from 0 to 0.777/0.776 (Acro205/Acro201). Among these, 39 exhibited high PIC values, ranging from 0.77 (Acro205) to 0.50 (Acro220); 61 exhibited medium PIC value, ranging from 0.49 (Acro125) to 0.25 (Acro213); 31 with low PIC value, ranging from 0.24 (Acro33) to 0.05 (Acro124); and 14 with null PIC value (monomorphic markers), according to Botstein et al. [25] (see Additional file 2). From all loci analysed, 124 private alleles were detected in 74 EST-SSRs, with Acro205 being the marker with more private alleles (7 private alleles) (Table 2). The 145 novel designed EST-SSR primers, annealing temperature, product size, and corresponding primer pair sequences are listed in the additional file 3.

**Table 2:** Private alleles and frequency for each EST-SSR detected in macaúba palm samples

EST-SSR	Allele size	Frequency	EST-SSR	Allele size	Frequency
Acro2	229	0.059	Acro162	136	0.059
Acro10	112	0.029	Acro164	246	0.059
Acro10	115	0.029	Acro165	157	0.294
Acro14	258	0.059	Acro168	233	0.063
Acro14	264	0.059	Acro168	290	0.063
Acro15	106	0.059	Acro171	449	0.147
Acro29	266	0.529	Acro172	252	0.029
Acro32	145	0.118	Acro178	350	0.029
Acro34	339	0.059	Acro178	356	0.029
Acro36	203	0.300	Acro183	383	0.235
Acro36	176	0.033	Acro183	395	0.059
Acro36	185	0.033	Acro183	404	0.059
Acro36	221	0.033	Acro187	201	0.038
Acro39	401	0.235	Acro188	206	0.059
Acro39	350	0.118	Acro188	209	0.029
Acro41	254	0.029	Acro189	277	0.088
Acro49	314	0.067	Acro189	291	0.059
Acro49	386	0.033	Acro189	307	0.059
Acro52	358	0.118	Acro191	243	0.059
Acro52	346	0.059	Acro192	358	0.059
Acro58	422	0.059	Acro193	300	0.059
Acro62	147	0.423	Acro195	397	0.067
Acro62	240	0.077	Acro196	253	0.063
Acro62	246	0.077	Acro198	280	0.067
Acro63	375	0.063	Acro199	349	0.059
Acro64	181	0.029	Acro199	352	0.059
Acro69	400	0.029	Acro201	354	0.179
Acro82	164	0.059	Acro201	342	0.036
Acro84	124	0.088	Acro201	369	0.036
Acro92	409	0.735	Acro201	417	0.036
Acro93	189	0.059	Acro203	215	0.206
Acro97	305	0.176	Acro203	167	0.118
Acro99	262	0.235	Acro203	260	0.029
Acro101	289	0.706	Acro205	120	0.094
Acro102	277	0.059	Acro205	126	0.063
Acro103	384	0.059	Acro205	171	0.063
Acro108	236	0.059	Acro205	180	0.031
Acro111	166	0.107	Acro205	183	0.031
Acro111	169	0.107	Acro205	192	0.031
Acro111	154	0.071	Acro205	195	0.031
Acro116	385	0.059	Acro206	338	0.250
Acro118	324	0.059	Acro206	362	0.063
Acro118	339	0.059	Acro206	365	0.063
Acro118	378	0.059	Acro206	385	0.063
Acro118	358	0.029	Acro208	409	0.471
Acro125	223	0.059	Acro208	418	0.059
Acro126	350	0.059	Acro209	213	0.529
Acro130	283	0.059	Acro210	242	0.133
Acro136	160	0.059	Acro210	278	0.033
Acro137	146	0.033	Acro212	242	0.176
Acro142	393	0.067	Acro212	170	0.059
Acro143	126	0.059	Acro212	113	0.029
Acro144	175	0.059	Acro212	152	0.029
Acro144	211	0.059	Acro213	239	0.059
Acro146	173	0.088	Acro213	236	0.029
Acro147	273	0.125	Acro217	105	0.059
Acro153	348	0.500	Acro218	142	0.059
Acro153	345	0.346	Acro218	109	0.029
Acro153	342	0.077	Acro223	212	0.059
Acro153	306	0.038	Acro227	369	0.059
Acro153	351	0.038	Acro227	390	0.059
Acro159	354	0.059	Acro228	259	0.265

**Fig. 1:**
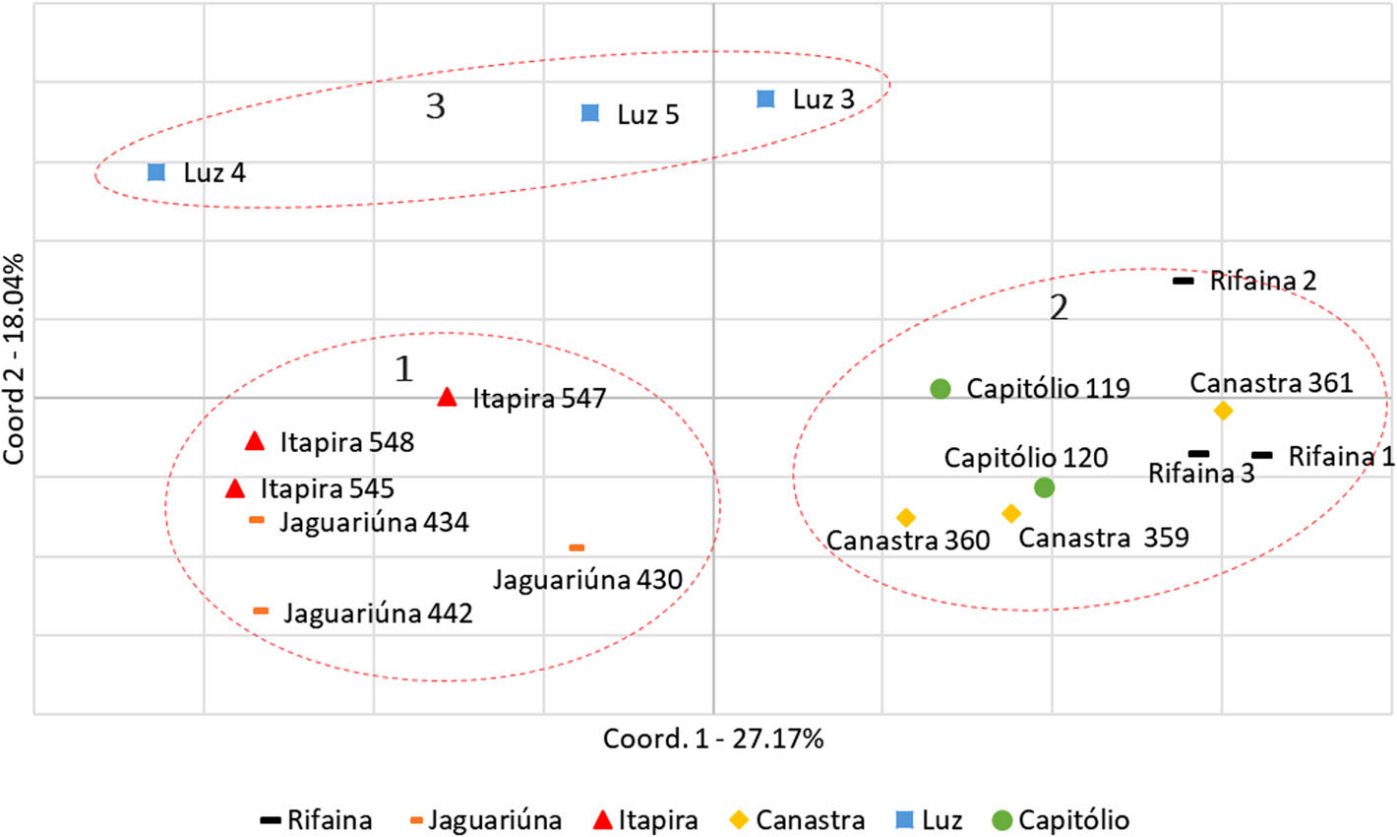
***Acrocomia aculeata*** **PCoA analysis.** Two-dimensional plot of principal coordinate analysis (PCoA) based on the Nei distance of *Acrocomia aculeata* samples from different locations for the 20 more discriminating EST-SSRs

Based on PIC values and probability of identity (PI) for all EST-SSRs, the most informative EST-SSR markers were selected to verify efficiency in the genetic analysis of the samples (additional file 4). Principal coordinate analysis (PCoA) was performed on the genotype data of 17 samples of macaúba palm based on the Nei distance [26]. The first and second axes explained 27.17 and 18.04% of the variation observed, respectively (Fig. 1). The PCoA results revealed three distinct genetic groups in accordance with their geographic location, containing samples from Itapira and Jaguariúna city from São Paulo State/Brazil (Group 1); Rifaina from São Paulo State/Brazil, Serra da Canastra, and Capitólio from Minas Gerais State/Brazil (Group 2); and Luz (Minas Gerais State/Brazil) (Group 3).

### Cross-transferability

The 145 EST-SSR primers from macaúba palm were examined for cross transferability in six species belonging to the Arecaceae family, including *Acrocomia totai* and *Acrocomia intumescens*. We observed a frequency of cross transferability of 99% in *Acrocomia intumescens* (144 EST-SSRs), 98% for *Acrocomia totai* (143), 80.7% (117 EST-EST) for African oil palm (*Elaeis guineensis*) and peach palm (*Bactris gasipaes*) samples, 70% (102) for the juçara palm (*Euterpe edulis*), and 71.7% (104) for the hat palm (*Sabal causiarum*).

Considering all parameters of descriptive genetics, the number of alleles ranged from 0 (when the EST-SSR was not transferred in the palm species) to 8 alleles (*Acrocomia totai*, EST-SSR 42). The juçara palm presented the lower means, and *Acrocomia totai*, the highest means for an effective number of alleles per locus (Ne) (1.193/1.936), mean expected heterozygosity (He) (0.189/0.367), mean observed heterozygosity (Ho) (0.102/0.204), and Shannon’s information index (I) (0.328/0.629) (see in Additional file 5).

**Fig. 2:**
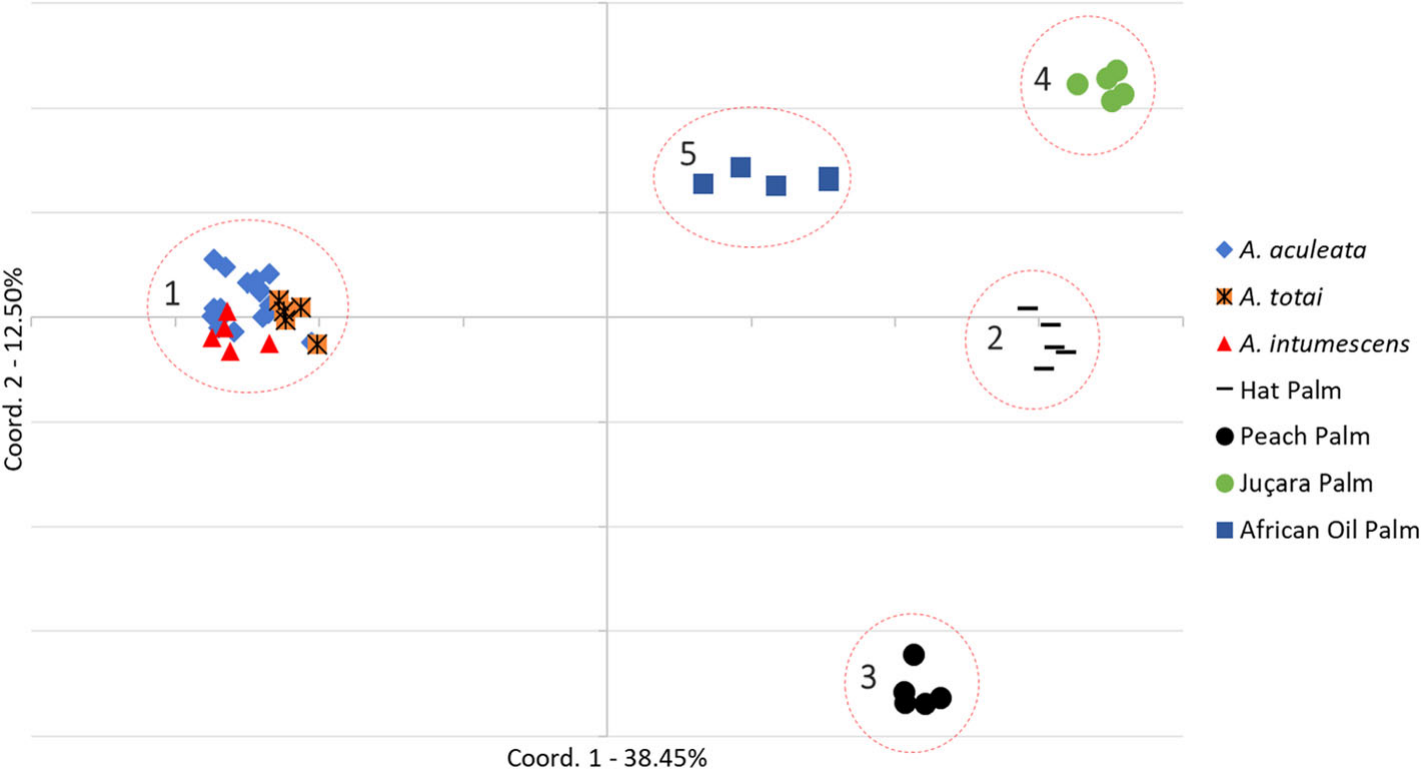
**Arecaceae family PCoA analysis.** Two-dimensional plot of principal coordinate analysis (PCoA) based on the Nei distance of Arecaceae samples for 145 EST-SSR data

The principal coordinate analysis (PCoA) performed on all sample with the 145 EST-SSRs (Nei distance [26]) explained 50.95% of the variance in the first and second axes with clear distinction of species and genera, which were structured in different groups (1 to 5) (Fig. 2). Non-amplified EST-SSR loci were considered as missing data.

## Discussion

As the next-generation DNA sequencing is becoming quicker and cheaper, vast amounts of sequence data are currently being generated exponentially, including a large number of ESTs from different plant species. Our study is the first to provide effective and useful markers from transcriptome analysis of 8 distinct tissues of macaúba palm of different individuals using next-generation sequencing, from which we identified and validated 145 macaúba palm EST-SSRs.

The macaúba palm EST database harboured highly abundant SSR sites. These EST-SSR markers proved to be useful for prior population structure analysis and will facilitate macaúba palm breeding, as well as studies with other palm trees with economic and environmental value upon transfer of these markers to these trees. In this study, 85.014 redundant EST-SSRs were identified in all samples, with a frequency of 1/1 Mbp, to prevent the validation of more than one locus in linkage equilibrium, according to assembly in the African oil palm genome. Although the density of the markers was lower than that obtained in other studies with pigeon pea (1/8.4 Kbp) [27], robusta coffee (1/3.4 Kbp) [28], caston bean (1/1.77 Kbp) [29], *Crambe abyssinica* (1/11.1 kb) [30] and *Cocos nucifera* (1/7.59 Kbp) [31], several useful markers were validated for use from the macaúba palm and other Arecaceae species. The density of SSR in EST depends on the search tool used, criteria used to identify SSRs in the database or redundancy in the set of SSR, which can lead to the identification of multiple markers at the same locus [15].

To date, in the macaúba palm, a small number of molecular markers have been available and validated [32], and they have been used in recent studies [13, 33–36]. Recently, 221 SSRs were identified in the chloroplast in a high frequency of occurrence (total mean of 1/1.75 Kb) located in intergenic spacers, coding regions, introns and tRNA genes; however, they have not been validated for the macaúba palm [37].

Our data revealed that trinucleotide repeats were the most frequent EST-SSRs, as observed by Zhao et al. [18] and Xia et al. [31] in the date palm and coconut palm, respectively. This suggests a result of selection and evolution because tri- and hexa-SSRs do not change the coding frame of the gene regions. Other repetitions change the frame and induce negative mutation when there is variation in SSR length [29].

The most frequent motifs were GAG/CAG, CCT/CCG and GGA/GGC, which encode glutamic acid, proline, and glycine, respectively. Among the proteins containing 10 or more repetitions of single amino acid, glutamine, alanine, glycine, glutamic acid, and serine repeats were more frequent than other amino acids [38, 39]. Katti et al. [40] observed that codon repeats corresponding to small hydrophilic amino acids are possibly more easily tolerated, and selection pressure possibly eliminates codon repeats encoding hydrophobic and basic amino acids. Consequently, the high level of occurrence of these motifs is substantial because amino acids produced by them are observed to a high extent in proteins.

Of the 418 EST-SSRs identified in different transcripts of all tissue samples, 145 (62.5%) markers produced clear bands. This rate is higher than that reported in the rubber tree (50%) [41], alfalfa (30%) [42], and adzuki bean (59.2%) [43] but lower than that reported in the castor bean (81.2%) [29] and mung bean (65%) [44]. The 87 primer pairs failed to generate the expected amplicon size or non-amplification PCR product, which may be due to the presence of introns and indels, since we have used the oil palm genome as a reference rather than the macaúba genome, lack of specificity or assembly error.

Although genic SSRs generally exhibits PIC values lower than that of genomic markers in population and diversity studies, we determined markers with high PIC values (> 0.5) [25], which were effective in discriminating the samples of the macaúba palm. The PIC values of our markers are higher than the values of the markers published by Nucci et al. [32] for the macaúba palm and are more valuable for marker-assisted selection and other applications.

The transferable nature of EST-SSR markers within related species or genera extends their usefulness in plant breeding and genetic studies, being limited in those species that have no available EST data set. We have achieved a high cross-species transferability of EST-SSRs found in the macaúba palm to other palm genera. It is advantageous to save time and cost for developing SSR markers for species that have not been largely studied. We obtained a transferability rate of 99% in *Acrocomia intumescens* (144 EST-SSRs), 98% for *Acrocomia totai* (143), and a high level of transferability in other palm trees.

The EST-SSR markers developed from *A. aculeata* offer a feasible solution for both correlational research of other related species that lack molecular markers and genetic studies in the *Acrocomia* genera. As demonstrated using the PCoA (Fig. 2), the Acrocomia genera was clustered into the same groups, and other palm species were clustered individually.

In the Arecaceae family, Zaki et al. [23] observed a high transferability level in Arecaceae members; 100% of *Elaeis oleifera* genomic SSRs were transferred to *Elaeis guineensis*, which are members of the same genre, and transferability in *Euterpe oleracea* (acai palm) was 72.7%, in *Oenocarpus multicaulis* was 63.6%, in *Jessinia bataua* was 54.5%, in *Ptychosperma macarthurii* (Macarthur palm) was 54.5%, in *Dictyosperma album* (Princess palm) was 45.5% and in *Cyrtostachys renda* (Lipstick *Palm*) was 45.5%. We obtained the same frequency of cross-transferability at the genera level as using genic SSRs (99 and 98%, respectively, for *A. totai and A. intumescens*/100% *Elaeis* sp.). Additionally, Mengistu et al. [24] observed that 44% of the markers developed by Zaki et al. [23] successfully amplified the genomic DNA in *A. aculeata*, of which 26% were polymorphic. Compared to genic SSRs, EST-SSRs are highly transferable at the genus, tribe and subfamily levels because of their location in conserved genic regions.

The strategy of obtaining EST-SSRs from reference-based transcriptome proved to be efficient since the SSRs identified were useful in separating the species and individuals within the *Acrocomia aculeata* species, as revealed in the principal coordinate analyzes. It is possible that some of EST-SSRs were lost and they could be capable of discriminate species of macauba palm and African oil palm, but since there is no macauba genome, the African oil palm reference was essential and useful to map regions with variable EST-SSRs.

We identified 418 EST-SSRs in all tissues; of these, 145 were amplified in *A. aculeata* samples, and a total of 100 polymorphic primer pairs were successfully amplified fragments, thus revealing abundant polymorphism between 17 *A. aculeata* samples. Additionally, of those 145 EST-SSRs, 144 were transferable in *Acrocomia intumescens*, 143 in *Acrocomia totai,* 117 in the African oil palm (*Elaeis guineensis*) and peach palm (*Bactris gasipaes*), 106 in the juçara palm, and 105 in the hat palm (*Sabal causiarum*), indicating that these newly developed EST-SSRs can be used with confidence in future population genetic studies of the 6 related species.

## Conclusion

This study has identified the wide occurrence of microsatellites in *Acrocomia aculeata*. The use of reference-based transcriptomic data analysis of different tissues of the macaúba palm for microsatellite development has been shown to be promising, and we were able to increase the number of useful EST-SSRs as a valuable sequence resource in both *A. aculeata* and the Acrocomia genus. The EST-SSRs reported in this study can potentially be a useful genomic tool in addition to other published SSR, as they provide a potential resource for association mapping of genera-related species. These EST-SSR markers have proven to be useful for both genetic mapping and population structure analysis, facilitating crop breeding of the macaúba palm, as well as studies with other palm trees with economic and environmental value.

## Methods

### Plant material and RNA isolation

The present study was performed using the tissues of leaves, leaf sheaves, roots, bulbs, fruit (mesocarp and endosperm) and male and female flowers from the macaúba palm (*Acrocomia aculeata*) for RNA isolation and transcriptome sequencing.

Vegetative tissues were collected from eight-month-old seedlings from native plants of Dourado, Amparo and Pedreira from São Paulo State/Brazil. The plants were acclimated for one month in a greenhouse. The male and female flowers were collected from adult plants at the Experimental Unit Santa Elisa – IAC (Agronomic institute of São Paulo/ Campinas/ Sao Paulo State/Brazil). The flower bunches were removed from the base to avoid damage to these materials. The fruit tissues (mesocarp and endosperm) were collected from Santo Antônio de Posse, Amparo from São Paulo State/Brazil, and Ibituruna from Minas Gerais State/Brazil at two fruit development times (developing fruit and ripe fruit).

RNA was isolated using the lithium chloride method [45, 46]; for fruit tissues, RNA was isolated using the perchlorate protocol [47]. Total RNA was treated with RNase-free DNase I (Takara, Kyoto, Japan) for 30 min at 37 °C to remove residual DNA. The RNA quality was verified using a 2100 Bioanalyzer (Agilent Technologies, Santa Clara, CA) and RNase-free agarose gel electrophoresis. The concentration of the total RNA was further quantified using an RNA NanoDrop (Thermo Fisher Scientific Inc., Waltham, MA, USA).

### Illumina sequencing, data filtering and reference-based assembly

The mRNA libraries were synthesized using the TruSeq Stranded mRNA Library Preparation Kit and sequenced using the Illumina paired-end sequencing technology (HiSeq2000). Prior to assembly, the 100 bp paired-end reads were submitted to quality filtering and adapter trimming using the Trimmomatic software version 0.36 [48]. All reads with more than 10% of bases with a poor-quality score (Q < 20) or non-coding RNA, as well as ambiguous sequences containing an excess of “N” nucleotide calls or adaptor contamination, were removed. Subsequently, the trimmed reads were mapped against the African oil palm genome [49] using TopHat version 2.0.13 [50] configured to allow up to two mismatches and three indels. The reference-based transcriptome assembly for each individual was performed using Cufflinks version 2.2.1 [51], merging all respective tissues.

### EST-SSR search and primer design

The MISA software [52] was employed for scanning EST-SSRs in the transcriptome assembly for each individual. The parameters were adjusted for the identification of perfect mono-, di-, tri-, tetra-, penta- and hexanucleotide motifs with a minimum of 10, 6, 5, 5, 5 and 5 repeats, respectively, and with a maximum distance of 100 bp between two SSRs.

SSR marker primer pairs were designed from the flanking sequences, using the PRIMER3 software [53], with the major parameters for primer lengths of 16–22 bases, GC content of 40–60%, annealing temperature of 50–60 °C, and PCR product size of 100 to 500 bp. To avoid amplicon size deviation generated by the presence of introns during DNA amplification, only PCR products located inside single exons were considered.

For the selection of unique and non-redundant SSRs in one transcript and region, the package BEDTools Version 2.26.0 was applied [54], with an intersection option that checked the overlap between all generated SSRs per individual. The chosen representative SSR was selected based on the nucleotide repeat size, melting temperature of forward and reverse primer, and motif type.

### Marker validation

Seventeen *Acrocomia aculeata* (macaúba palm) plants were selected according to the geographic location for polymorphism investigation of the EST-SSRs (Table 3). DNA isolation from young leaf tissues was conducted according to the CTAB DNA extraction protocol [55]. The quality and quantity of DNA were evaluated on a 1% agarose gel using the NanoVue™ Plus Spectrophotometer (GE Healthcare). Contamination with phenol/carbohydrates and proteins was measured based on optical density A260/A230 and A260/A280, respectively.

**Table 3:** Macaúba palm samples used for EST-SSR validation and genetic analysis

Species	Plant	Local
*Acrocomia aculeata*	Plant 1	Rifaina -SP
*Acrocomia aculeata*	Plant 2	Rifaina -SP
*Acrocomia aculeata*	Plant 3	Rifaina -SP
*Acrocomia aculeata*	Plant 430	Jaguariúna - SP
*Acrocomia aculeata*	Plant 434	Jaguariúna - SP
*Acrocomia aculeata*	Plant 442	Jaguariúna - SP
*Acrocomia aculeata*	Plant 545	Itapira - SP
*Acrocomia aculeata*	Plant 547	Itapira - SP
*Acrocomia aculeata*	Plant 548	Itapira - SP
*Acrocomia aculeata*	Plant 359	Serra da Canastra
*Acrocomia aculeata*	Plant 360	Serra da Canastra
*Acrocomia aculeata*	Plant 361	Serra da Canastra
*Acrocomia aculeata*	Plant 3	Luz – MG
*Acrocomia aculeata*	Plant 4	Luz – MG
*Acrocomia aculeata*	Plant 5	Luz – MG
*Acrocomia aculeata*	Plant 119	Capitólio - MG
*Acrocomia aculeata*	Plant 120	Capitólio - MG

PCR reactions were performed in a 15 μL total volume containing 20 ng of template DNA, 2.0 μL of each forward and reverse primers (5 μM/μL), 3 μL of Hot Start PCR Master Mix (2X) and 8.2 μL of ultrapure water. PCR amplifications were performed in a thermal cycler (T100 - Bio-Rad) as follows: initial denaturation at 94 °C for 2 min, followed by 30 cycles at 94 °C for 1 min, 55–58 °C (depending on the primers requirement) for 1 min, 72 °C for 1 min and a final extension at 72 °C for 10 min. The amplification products were separated by capillary electrophoresis using a Fragment Analyzer™ 96-capillary Automated CE System (Advanced analytical Technologies, Ames, IA, USA) using the DNF-905 double-stranded DNA Reagent Kit (Advanced Analytical Technologies, Ames, IA, USA). For this analysis, 5 μL of each amplification product was diluted in 19 μL of buffer and placed in 96-well microplates.

### Cross-species SSR transferability

It is well known that the genic regions are highly conserved and provide a cost-effective source of markers for related species, which is especially important for taxa with low microsatellite frequencies or from which microsatellites are difficult to isolate.

In this study, we investigated the transferability of genic SSRs with five plants of six different species, which have been selected based on leaf samples. *Acrocomia totai, Acrocomia intumescens*, the hat palm (*Sabal causiarum*), the juçara palm (*Euterpe edulis*) and the peach palm (*Bactris gasipaes*) have been collected from the palm trees of the Botanical Garden of the Agronomic Institute of Campinas. Samples of the African oil palm (*Elaeis guineensis*) were collected from the germplasm (genotypes BRS C2328, BRS C2528, BRS C7201, BRS C2001 and Manicoré hybrid). Genomic DNA isolation and PCR amplification were performed as described above.

### Genetic diversity analysis

Estimates of expected heterozygosity (He), observed heterozygosity (Ho), effective number of alleles (Ne), polymorphism information content (PIC), probability of identity (PI), private alleles per locus, and Shannon’s information index (I) were calculated using the software GenAlEx 6.5 [56, 57]. Principal Coordinate analysis (PCoA) was conducted using the software package GenAlEx based on Nei distance [26].

## Additional files

Additional file 1: **Table S1.** Number and frequency of SSR motif and type of motif of 418 EST-SSRs identified in the macaúba palm transcriptome data. (XLSX 11 kb)

Additional file 2: **Table S2.** Descriptive statistics of 145 EST-SSR markers validated in the macaúba palm. (XLSX 20 kb)

Additional file 3: **Table S3.** Validation of 145 EST-SSR primer sequences in macaúba palm samples and their transfer to palm tree samples. (XLSX 24 kb)

Additional file 4: **Table S4.** Twenty EST-SSR that are more informative, primer sequence, and genetic statistics. (XLSX 12 kb)

Additional file 5: **Table S5.** EST-SSR transferred in *Acrocomia totai*, *Acrocomia intumescens*, hat palm, peach palm, juçara palm, and African oil palm. (XLSX 41 kb)

## Abbreviations

*EST-* SSR: *Expressed Sequence Tag* - *Simple* Sequence Repeat
He: Expected heterozygosity
Ho: Observed heterozygosity
I: Shannon’s information index
Ne: Effective number of alleles per locus
PCoA: Principal coordinate analysis
PI: Probability of identity
PIC: Polymorphism information content
